# Biology, Ecology, and Pest Management of the Tarnished Plant Bug, *Lygus lineolaris* (Palisot de Beauvois) in Southern Row Crops

**DOI:** 10.3390/insects12090807

**Published:** 2021-09-09

**Authors:** Justin George, James P. Glover, Jeffrey Gore, Whitney D. Crow, Gadi V. P. Reddy

**Affiliations:** 1 USDA-ARS, Southern Insect Management Research Unit, Stoneville, MS 38776, USA; James.Glover@usda.gov (J.P.G.); Gadi.Reddy@usda.gov (G.V.P.R.); 2 Research & Extension Center, Mississippi State University, P.O. Box 197, Stoneville, MS 38776, USA; JGore@drec.msstate.edu (J.G.); wdc165@msstate.edu (W.D.C.)

**Keywords:** tarnished plant bug, *Lygus lineolaris*, cotton, *Lygus* management, insecticide resistance in cotton insects, Bt cotton

## Abstract

**Simple Summary:**

The tarnished plant bug, *Lygus lineolaris*, is a polyphagous, sap-feeder that causes significant economic damage in several field crops, especially cotton (*Gossypium hirsutum* L.) in the mid-southern United States. In 2020, it was reported that 4.8 million acres of cotton were infested by *Lygus* spp. in the United States. A broad host range, polyphagous feeding behavior and high mobility of this pest along with resistance development to conventional pesticides helped them establish as a significant pest of concern for cotton growers in the mid-south. Since the publication of a review by Layton (2000) on damage caused by *Lygus lineolaris*, many new research studies have been published on the *Lygus* biology, ecology, and integrated pest management strategies. A comprehensive review paper that summarizes these latest research developments and *Lygus* management strategies will be useful for researchers and cotton growers. In this review, we report and discuss the latest developments in *Lygus* research and the new control strategies that have been developed in the last two decades.

**Abstract:**

The tarnished plant bug, *Lygus lineolaris* (Palisot de Beauvois), (Hemiptera: Miridae) is considered the most damaging pest of cotton (*Gossypium hirsutum* L.) in the mid-southern United States, although it is established throughout the United States, southern Canada, and northern Mexico. The introduction of transgenic crops for the control of moths in the *Heliothine* complex and eradication of the boll weevil, *Anthonomus grandis*, from much of the United States led to greatly reduced pesticide use in cotton fields, which allowed *L. lineolaris* to emerge as a new primary pest of cotton in the mid-southern United States. Since the publication of a review by Layton (2000) on damage caused by *Lygus lineolaris*, many new studies have been published on the changes in host range, population dynamics, sampling methods and thresholds, cultural practices, sex pheromones and attractant blends, novel pesticides and insecticide resistance mechanisms, olfactory and feeding behaviors, introduction of biological control agents, host-plant resistance mechanisms, and new molecular and genetic tools for integrated pest management of *Lygus* species in cotton and other important crops. Here, we review and discuss the latest developments in *L. lineolaris* research in the last two decades.

## 1. Introduction

The success of boll weevil (*Anthonomus grandis*) eradication program and the introduction of transgenic (Bt) cotton in the mid-1990s considerably reduced the application of broad-spectrum insecticides in US cotton fields. Consequently, the tarnished plant bug, *Lygus lineolaris* (Palisot de Beauvois), (Hemiptera: Miridae), which previously had been indirectly controlled by the applications of broad-spectrum insecticides targeting other cotton pests, emerged as a major pest of cotton and other crops in the USA. *L. lineolaris* is a polyphagous, sap-feeding true bug that causes significant damage in several crops, especially cotton (*Gossypium hirsutum* L.) in the mid-southern United States. *L. hesperus* (Knight) and *L. elisus* (Van Duzee) are the other major species of pest *Lygus* found in agricultural crops in North America. *L. lineolaris* is the most widely distributed *Lygus* species in North America and is reported from Mexico to Alaska [1]. It feeds on over 700 plant species in 55 families in North America, including 130 crops of economic importance [2], including cotton, seed alfalfa, snap and lima beans, soybeans, apples, cherries, strawberries, pears, peaches, tomatoes, carrots, and nursery stock [3,4]. In Canada, it is a major pest in various vegetable crops and in fruit crops such as grapes and strawberries [5]. The population dynamics of this insect pest are affected by the spatial distribution of economically important crops and weedy host plants in a region’s agroecosystem, and many of the mirid’s host plants are broad-leaved dicots [6]. In the southern United States, two key winter and spring host plants are shepherd’s purse, *Capsella bursa-pastoris* (L.) Medicus, and henbit, *Lamium amplexicaule* (L.), and these species are important overwintering sites of *L. lineolaris* since they bloom from December through April [7]. The abundance of *L. lineolaris* is greater in cotton fields that are adjacent to other crops or natural ecosystems [8], and *Lygus* bugs’ movement is affected by plant diversity in the local agroecosystems and by the bugs’ long-range dispersal ability. Once the early season host plants have senesced, adult *L. lineolaris* move into cotton [9]. Stewart and Gaylor [10] observed *L. lineolaris* to fly more than 12 km in 12 h, and this flight ability makes this species an efficient polyphagous herbivore able to exploit many different agricultural crops.

*Lygus lineolaris* feeds on cotton squares and small bolls, which reduces lint quality and yield and causes millions of dollars in damage annually [11,12]. *L. lineolaris* can damage cotton throughout the growth of the crop, from emergence through the early lint development stage of the last harvestable bolls [11,13]. Yield reductions are attributed to the loss of first fruiting position bolls [14]. *Lygus* feeding injuries can also introduce boll rot pathogens, causing further lint and seed degradation [15]. The reduction in broad-spectrum insecticide applications in cotton fields, following the success of the boll weevil eradication program and the introduction of transgenic cotton (Bt cotton), contributed to the emergence of *L. lineolaris* as a major cotton pest [16]. In addition, development of *Lygus* resistance development to pyrethroid, organophosphate, carbamate, and cyclodiene insecticides [17] raised the importance of these bugs as cotton pests. By 2009, seven insecticide applications were being applied annually in the Mississippi Delta region (one of the major US cotton production areas) for control of *L. lineolaris*, at a cost of USD 175 ha^−1^ [18]. Similarly, in 2016, 71.3% of North Carolina cotton was sprayed for *L. lineolaris* control, with fields receiving on average three insecticide applications per acre annually. Moreover, nearly all Virginia cotton fields were sprayed for *L. lineolaris,* with an average of two insecticide applications per acre [19].

Damage caused by *L. lineolaris* in cotton was last reviewed by Layton [11] 20 years ago. Since that review, there have been many new studies of *Lygus* biology, ecology, host range, population dynamics, sampling thresholds, olfaction and feeding behaviors, sex pheromones and attractant blends, rearing methods, sampling methods and economic thresholds for insecticide applications, chemical control tactics, and IPM strategies for *Lygus* control in cotton and other crops. Recent studies also reported on the use of semiochemicals for trapping and monitoring, new cultural practices, introduction of biological control agents, development of new host plant resistance strategies based on the use of Cry proteins from *Bacillus thuringensis* (Bt), and the use of molecular tools and techniques to develop efficient management practices against different *Lygus* species. This review will report and discuss recent advances in the *L. lineolaris* research and the different management strategies reported in recent years.

## 2. Biology and Life Cycle

*Lygus lineolaris* (tarnished plant bug) is a true bug in the Miridae family that feeds with piercing-sucking mouthparts [20]. It has a large host range that includes non-crop plants, commercial flowering plants, nursery plants, fruit crops, vegetable crops, greenhouse crops, grains, and row crops. Over half of the cultivated plant species grown in the United States are listed as host plants for *L. lineolaris* [21], and it can also damage conifer seedlings near cultivated areas. In cotton, *L. lineolaris* feeds primarily on the meristematic tissues of cotton squares and small bolls, resulting in square abscission, damage to the anthers and the staminal column, and death of pinhead squares [22]. *L. lineolaris* adults are greenish to brownish in color with reddish-brown markings. Adults are 5–6 mm long (¼ inch) and 2.5–3 mm wide with a flattened body that is oval in outline. Adults have a characteristic light-colored “V” behind the head and two light-colored patches further back on the wings [23]. The summer adult color varies from pale yellow with few black markings to reddish brown, or almost completely black with few pale-yellow markings [1,24]. Overwintered adults are much darker than summer adults [21].

**Figure 1:**
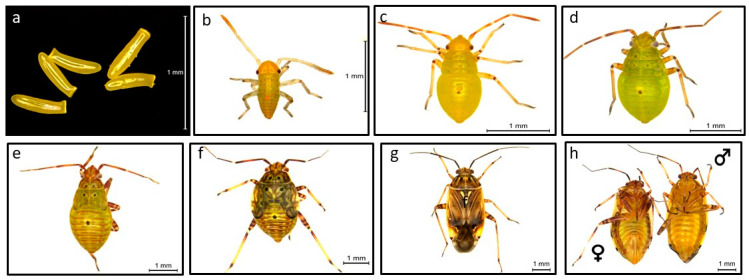
*Lygus lineolaris* (Hemiptera: Miridae) lifecycle stages. (**a**) Eggs; (**b**) 1st instar nymph; (**c**) 2nd instar nymph; (**d**) 3rd instar nymph; (**e**) 4th instar nymph; (**f**) 5th instar nymph; (**g**) adult; (**h**) ventral side of adult female with ovipositor (**left**) and adult male (**right**).

Eggs are inserted inside plant tissues (stems, petioles, midribs, buds, flower heads), but the preferred location varies with the plant host [21]. The egg (1 × 0.25 mm) is small, truncate, and slightly curved (Figure 1a). The top of the egg where it emerges from the surface of the plant tissue is flattened and has an opening through which the new nymph emerges [21]. Depending on the temperature, eggs take 7–12 days to hatch into small greenish-yellow first instar nymphs (1 mm long) (Figure 1b). At the optimum temperature for oviposition (20 °C), each female lays an average of 96 eggs, but may lay as many as 140. There are five nymphal instars (Figure 1b–f), each about 3–4 days in duration. The fourth and fifth instars have four black spots on the thorax and one on the abdomen (Figure 1e,f). Fifth-stage nymphs (4.5 × 2 mm) have visible wing buds [25] (Figure 1f). The whole life cycle takes 30–40 days. The female’s abdomen is more pointed in shape with the ovipositor (Figure 1g,h), and they live for about 52 days at 20 °C, and 37 days at 28 °C [1]. The intensity and extent of the color pattern, and differences between males and females, are probably influenced by temperature, humidity, sunlight, host plant, and age of the individuals [1]. *L. lineolaris* bugs have several generations per year in the mid-southern states such as Mississippi, Arkansas, Louisiana, Alabama, Southeast Missouri, and Tennessee. The first several generations are completed on wild, non-crop plants that mature in the early season [26]. Subsequent generations move into cotton or other crops where they cause significant economic damage [27]. *Lygus lineolaris* overwinter as adults in leaf litter, under bark, or between leaves of herbaceous plants or dry grass. Diapause is induced when nymphs are exposed to short days (<12.5 h) and resultant adults enter diapause. However, exposure to continuous light prevents diapause development in young adults and terminates diapause in adults already in diapause.

## 3. Host Plants, Crop Damage, and Factors Promoting Outbreaks

### 3.1. Distribution and Host Plants

*Lygus lineolaris* is a highly polyphagous mirid that damages many economically important plants across much of the Americas and is found in all the agricultural regions of the United States [28]. The species is recorded from Alaska, east Newfoundland, and through Central America into South America [11]. It is among the most damaging of the mirids and has the broadest diet breadth of any arthropod [28,29]. This wide geographic distribution and large number of non-crop hosts across the continental United States has contributed to this insect’s pest status. *L. lineolaris* is known to feed on up to 700 cultivated or non-cultivated plant species, especially broadleaf, herbaceous plants [4,7,29]. *L. lineolaris* utilize hundreds of plant species as food sources with the major hosts from the subclasses Rosidae and Asteridae [4,7]. In the Mississippi River Delta region (i.e., Arkansas, Louisiana, Mississippi) 169 host plant species from 35 families have been reported, most of which are herbaceous non-crop plants. The most frequently reported hosts plants are from the Asteraceae, Fabaceae, Polygonaceae, Brassicaceae, Amaranthaceae, and Onagraceae, with fewer host plants found in 30 more families [7]. In the Mississippi Delta region, adults can be collected from wild host plants in every month of the year. However, the sequence use of host plants can be affected by annual precipitation and temperatures [30,31]. From early January through March, primary winter and spring hosts include species of Brassicaceae, Fabaceae, Polygonaceae, and Onagraceae, while fall host plants (September through December) are mainly in the Amaranthaceae and Polygonaceae. Plants in the Asteraceae are used from early March through December.

The most important non-crop wild hosts of *L. lineolaris* populations in the mid-southern United States are tall goldenrod (*Solidago altissma* L.), pigweed (*Amaranthus* spp.), pinkweed (*Polygonum penslyvanicum* L.), white heath aster (*Aster pilosus* Willdenow), and daisy fleabane (*Erigeron* spp.) [7,29]. In the winter months (late December through March) when temperatures range from 47–58 °F or above, there are two main weedy wild plants that bloom widely across the Mississippi Delta, namely henbit (*Lamium amplexicaule* L.) and shepherd’s purse (*Capsella bursa-pastoris* L. Medicus). However, sour dock (*Rumex crispus* L.) (Polygonaceae) is the only host plant on which *L. lineolaris* is found throughout the whole year in the Mississippi Delta. The presence of flowering vegetation is key to *L. lineolaris* attraction and reproduction, as flowers are the preferred feeding site [7]. All the above-mentioned wild hosts are found along field margins and roadside ditches in great abundance, and many of these species grow in dense stands that can support large *L. lineolaris* populations. In cotton agroecosystems, *L. lineolaris* is well documented to occur on many species of weeds in field margins and then migrate into cotton fields once the weedy hosts senesce or are destroyed [2,7,8].

### 3.2. Crop Damage

Historically, *L. lineolaris* has been considered a pre-bloom pest in cotton, particularly affecting the plant during the first several weeks of squaring. However, the adults and nymphs can attack and damage cotton from plant emergence until the plant approaches physiological maturity. Typically, most economic damage from *L. lineolaris* in cotton occurs from the third week of squaring through the fourth week of bloom [32]. The indeterminate growth of cotton causes the full range of fruiting structures (i.e., squares, flowers, developing fruit) to be simultaneously present for nearly 2 months, allowing multiple generations of *L. lineolaris* to develop in the crop [32]. Fruit retention during the pre-bloom period is critical to maximize yield. Major economic losses primarily occur from damage to, (even complete loss of) the first fruiting position, which provides a significant proportion of the yield in cotton [14].

**Figure 2:**
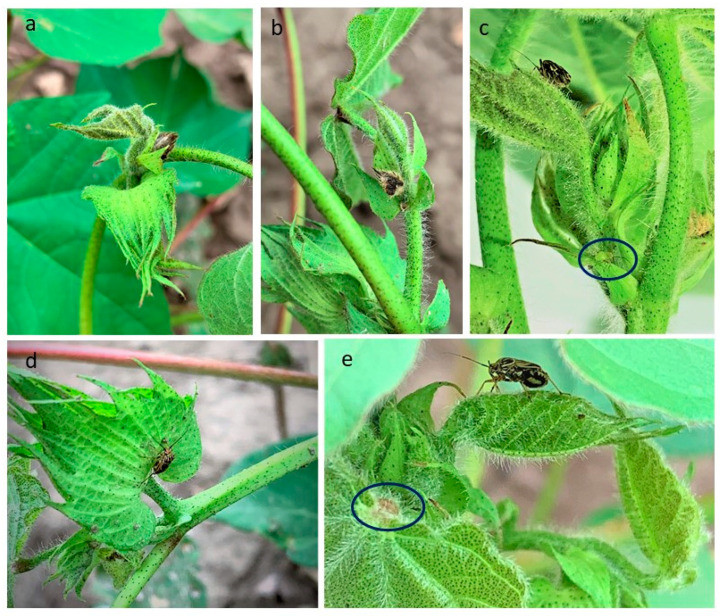
*Lygus lineolaris* feeding and the damage on cotton squares. (**a**,**b**) Aborted black cotton squares from *L. lineolaris* feeding; (**c**) *L. lineolaris* adult and early instar nymph (blue oval) on a cotton square; (**d**) *L. lineolaris* adult feeding on a cotton square; (**e**) *L. lineolaris* adult on a late-stage cotton square, and the feeding damage on an early-stage cotton square called “blasted square” (blue oval).

*L. lineolaris* feeding primarily occurs on the plant’s reproductive structures, where bugs insert their mouthparts, introducing saliva with foreign and toxic substances into the plant, and then extract plant liquids from developing fruits and seeds. They prefer to feed on small to medium sized flower buds (squares). Feeding damage to newly developing floral buds or squares may occur in a range from small to medium sized fruit (Figure 2a–d) [32]. When fruiting structures are small enough (i.e., pinhead) and depending on the duration and severity of feeding the square will abscise or abort [33]. A small scar will mark the abscised fruiting site and is colloquially referred to as a “blasted square” (Figure 2e). When feeding occurs on larger fruiting structures (i.e., larger developing squares), the fruit are often retained but may have various types of external and internal damage. Larger squares that are retained but have sustained feeding or probing damage can result in “dirty blooms,” with yellow staining on the external surface of the square and black or brown discolored anthers on the subsequent flower. More than 30% anther damage in developing flowers can lead to malformed or abscised bolls, causing yield loss [11]. Green et al. [34] demonstrated that they may also damage bolls up to 8 days old but that yield loss is negligible after bolls have accumulated 250–300 heat units (degree-days [DD]) after flowering.

The close association between cotton boll-rot diseases and boll-feeding by piercing-sucking insects is well documented [35,36]. *L. lineolaris* is known to transmit disease pathogens, and the infected bolls that do mature often have misshapen fruit or malformed bolls and locules. Damaged bolls that are retained on the plant may exhibit a disease known as “hardlock”, which is often associated with the boll-rot disease complex [37]. Affected bolls often exhibit reduced seed weights, and fibers fail to mature and elongate properly, further reducing yield [37]. External damage is most often restricted to the specific locule where feeding took place (visible as feeding punctures). At puncture sites, small, black, sunken lesions may develop externally that penetrate the plant tissue and extend through the carpel wall causing lint deterioration and seed necrosis [34]. Feeding injuries can also introduce various boll-rot pathogens that cause further lint and seed degradation [15]. *L. lineolaris* can also feed on apical tissues, resulting in irregular, stunted growth that significantly delays crop maturity and harvest [32,34]. Their feeding increases vegetative growth (i.e., non-sympodial branches) and lengthening of internodes, leading to loss of apical dominance of plant terminals. *L. lineolaris* feeding during the early weeks of vegetative growth of flowering structures can lead to abnormally tall plants with poor fruit set and lower fruit retention, sometimes referred to as “crazy-cotton” or “bushy top” [38].

Extensive research has been carried out over the last two decades towards describing plant response to plant bug damage, and more specifically defining the window of susceptibility to developing cotton plants. *L. lineolaris* damage has been shown to occur on a wide range of reproductive and fruiting structures particularly during late squaring through the first 3 weeks of bloom. Floral buds or squares when fed can abscise, while more mature squares that develop into bolls can manifest damage cosmetic in nature with no internal damage or harvestable loss. The window of vulnerability for developing cotton bolls to *L. lineolaris* damage is significantly less after bolls accumulate 250–300 heat units (degree-days [DD]) or 8 days post-anthesis when the carpel wall has become sufficiently thick and mature that probing activity is less damaging. Future research avenues should evaluate modern and commercially available cotton cultivars and varieties to benchmark and compare plant response to herbivory under varying environmental conditions. This may help partition abiotic stressors such as unfavorable growth conditions from insect derived losses that impact current threshold dynamics.

### 3.3. Factors Promoting Lygus Outbreaks

**Figure 3:**
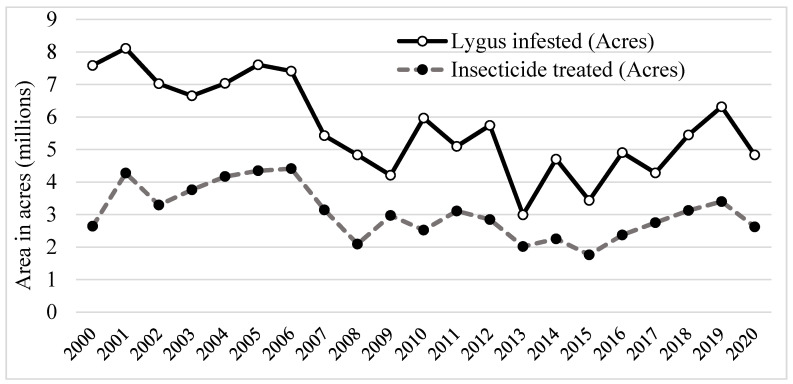
Total *Lygus* infested acreage and the insecticide treated acreage for *Lygus* control (millions) in the United States during the last 20 years. Cotton crop loss data https://www.biochemistry.msstate.edu/resources/cottoncrop.php (accessed on 22 June 2021).

*L. lineolaris* is one of the most widely distributed and economically recognized insect pests wherever it occurs and is the most damaging insect pest in cotton in the mid-South [16,32]. Their relative importance and outbreak status as a key pest of cotton has significantly increased over the last 20 years, and 4.8 million acres were infested in 2020 (Figure 3). This increase in its pest status has largely been due to several major changes in cotton insect and crop management strategies, including the advent and nearly global adoption of plant-incorporated protectants such as *Bacillus thuringiensis* (Bt) proteins, successful boll weevil eradication across the region (i.e., Mississippi, Alabama, Arkansas, Tennessee) [16], and the development of insecticide resistance in *L. lineolaris*. Snodgrass [39] first documented pyrethroid insecticide resistance in 1995, and by 1999 resistance had developed in *Lygus* populations throughout much of the mid-South [28]. Subsequently, acephate resistance was documented in *L. lineolaris* populations, conferring cross resistance to other organophosphates [17].

Historically, *L. lineolaris* was considered only a secondary pest of relatively minor importance [40,41] compared with the boll weevil or pesticide-resistant tobacco budworm (*Heliothis virescens* F.) populations. The first generation of genetically modified (GM) cotton such as Bollgard (Monsanto Co., St. Louis, MO, USA) became commercially available in 1996, providing cotton varieties resistant to lepidopterans, with specificity against the *Heliothine* complex. These varieties were followed by a second generation of resistant cotton varieties in 2003 (Bollgard II, Monsanto Co., St. Louis, MO, USA) and 2005 (Widestrike, Dow AgroSciences, Indianapolis, IN, USA) [32]. Currently, across the mid-south more than 80% of the transgenic cotton varieties that are planted in the region express two or more insecticidal proteins providing stacked or pyramided insecticide resistance traits. This development resulted in the elimination of many foliar applications to control pests of flowering cotton. These advances in development of resistant cotton varieties have greatly reduced the number of broad-spectrum insecticide applications in cotton, producing a low-spray environment allowing plant bugs to reach outbreak levels [32,42]. Reliance on foliar applications to control *Lygus* populations in the Bt era resulted in the widespread development of pyrethroid and organophosphate resistance throughout the mid-south, which in turn has affected current cotton pest management strategies [28,39,42]. Additionally, the use of seed treatments for control of early season pests such as thrips, aphids, and other foliar pests has gained widespread popularity, leading to a reduction of the use of traditional in-furrow applications of carbamates such as aldicarb. The resulting low-spray environment in cotton fields, coupled with the widespread use of seed treatments to control early season pests, combined with increasing insecticide resistance in *L. lineolaris* across multiple chemistries and modes of action explain the elevated pest status and seasonal outbreak pattern currently observed for *L. lineolaris* across the mid-southern United States.

Abiotic factors (temperature, humidity, and day length) are also important in promoting outbreaks of *L. lineolaris*. Diapause in *L. lineolaris* was first described as hibernation [43]. Laboratory investigations later demonstrated that this diapause was induced by exposure of nymphs to day lengths of 12.5 h or less [44]. Snodgrass [31] later estimated the date that the critical photoperiod for diapause induction in the Mississippi delta occurs by September 12. Other physical factors affecting *L. lineolaris* population densities include latitude and weather. Prolonged periods of cool weather may also slow the growth of populations of beneficial insects that attack *L. lineolaris* and thus indirectly contribute to sporadic *L. lineolaris* outbreaks that are sometimes observed across the Mississippi delta.

Future research should investigate the phenology and annual succession of weedy host plants that are utilized by *L. lineolaris,* as this may aid in the identification and management of potential population outbreak sources across the landscape. Additional investigation of the region’s predominant non-cultivated weedy hosts and their role in diapausing and non-diapausing populations of *L. lineolaris,* may help explain the periodicity and emergence patterns observed during spring and late summer populations when crops are unavailable, or have become less attractive. The effects of extreme temperatures such as heat waves and the potential for aestivation may provide additional resolution and improved predictive capability of potential outbreak populations, estimation of first emergence, and other climatic and landscape interactions that may contribute to population outbreaks.

## 4. Behavior and Ecology of *Lygus lineolaris*

### 4.1. Semiochemicals

Discovery and optimization of insect sex pheromones and other behavior modifying compounds are important in developing mass trapping and mating disruption strategies and have potential for use against plant bugs, which would be valuable given their seasonal distribution changes across different host plants. Even though many studies have reported some progress in the identification of mirid pheromones [45,46,47,48], very little is known about any such sex pheromones in *L. lineolaris.* Chinta et al. [49] reported the ultrastructure of the antennae of *L. lineolaris* adults, which consists of four-segmented regions that contain ~2000 sensilla hairs, corresponding to six sensillar types and representing sensilla trichodea, sensilla chaetica, and sensilla basiconica. Zhang and Aldrich [47] reported that the contents of the metathoracic scent gland (MTG) of female mirids can act as a sex pheromone. Follow up studies showed that both males and females of *L. lineolaris* produce these MTG compounds, which include hexyl butyrate, (E)-2-hexenyl butyrate, and (E)-4-oxo-2-hexenal. These compounds elicit antennal and behavioral responses from both sexes [50]. Blends of these MTG components, however, failed to attract either sex, but intact, live virgin females did attract significant numbers of males under field conditions [50]. Wardle et al. [51] reported three more additional compounds—(*E*)-2-hexenal,1-hexanol, (*E*)-2-hexenol—that are produced by disturbed male and female *L. lineolaris* and by calm females. The compound (*E*)-2-hexenal was lacking in volatiles collected from calm males. The repellent effects of these compounds were ephemeral and were reported to have no practical application as repellents for this hemipteran pest [51]. Zhang et al. [52] described the morphological structure of MTGs in the plant bug *Adelphocoris suturalis* (Jakovlev), and in GC-MS studies, they showed that hexyl butyrate (85%) was the major component of MTG secretions. These compounds from the MTGs of mirids may act as defense, sex, or aggregation pheromones, and they play an important role in intraspecific communication and sexual behavior [47,53,54]. In *Stenotus rubrovittatus* (Uhler) (the sorghum plant bug) and *Lygus rugulipennis* (Poppius) (the European tarnished plant bug), hexyl butyrate acts as a sex pheromone [55,56]. The compound (E)-2-hexenal is also present in secretion of the MTG of female *A. suturalis*, and it has been reported to be a defensive compound in ants, beetles, cockroaches, and true bugs [53]. Harraca et al. [57] reported that (E)-2-hexenal acts as an alarm pheromone in *Cimex lectularius* (L.).

Brent and Byers [58] reported that myristyl acetate acts as a male derived anti-aphrodisiac sex pheromone that is inserted into females by males of *Lygus* spp. during mating and repels males for a few days. Byers et al. [59] reported that the ratios of the two components of the *Lygus* spp. sex pheromone (made up of hexyl butyrate and E-2-hexently butyrate) varied between females of *L. lineolaris*, *L. elisus* (4:10), and *L. hesperus* (10:1). This difference in sex pheromone component ratios contributes to their function by preventing cross mating among these species. Fountain et al. [60] reported another attractive lure based on the volatiles produced by females of *Lygus* spp. Yang et al. [61] also studied the MTG components that act as sex pheromones in different mirid species and reported on the compounds’ role in intraspecific attraction and reproductive isolation among this group of species. Parys and Hall [62] tested different MTG components, in different ratios, in combination with white sticky cards under field conditions to attract *L. lineolaris.* An attractant lure expressing a ratio of 4:10:7 of hexyl butyrate, (E)-2-hexenyl butyrate, and (E)-4-oxo-2-hexenal was most effective in collecting *L. lineolaris*. The numbers of insects collected were similar to those collected using traps baited with virgin females of *L. lineolaris*.

*Lygus lineolaris* is also a major pest of fruits and vegetables and is reported to cause major damage to strawberries in Quebec, Canada [63] and other parts of the world. Chouinard-Thuly [64] studied the use of sticky traps baited with sunflower volatiles (48.9% pinene, 34.2% sabinene, and 16.9% phenylacetaldehyde) [65] and sex pheromones [59,62] for capturing *L. lineolaris* in strawberry fields in Quebec, Canada. This study reported a significantly lower catch of *L. lineolaris* in traps baited with sex pheromone + sunflower volatiles compared to the control traps. Moreover, Ondiaka et al. [65] reported a reduction in the response of *L. lineolaris* to sex pheromones when they were tested in combination with sticky traps. The release rates and the volatile background of strawberries may have affected the trap catch in this study. Even though the trap catch responses were not consistent with those reported for sex pheromones in previous studies, most of these studies have shown that a high abundance of (E)-4-oxo-2-hexenal is essential for attraction in *L. lineolaris* [50,51,59,64]. Even though a significantly greater number of *L. lineolaris* were attracted to these MTG blends compared to the controls, the mean number of insects collected in these traps were very low [62]. The color of the sticky cards used, the trap characteristics, and the location of trap placement may have caused this decreased trap catch in these reported studies. There are multiple *L. lineolaris* attractant blends reported from different cotton growing areas in the US, and these blends need to be further tested in a coordinated multistate trial to study its efficacy in attracting *L. lineolaris* at different locations. Further studies are needed to explore these long-range visual cues, and short-range olfactory cues that influence the behavior of *L. lineolaris* under field conditions. An attract-and-kill device based on these visual cues and MTG compounds may have practical application in monitoring and management of *L. lineolaris* under field conditions.

### 4.2. Visual Cues and Trap Characteristics That Affect Lygus lineolaris Captures

Visual cues play an important role in the host finding behavior of insects and understanding the roles of these visual cues is important in designing and development of visual traps for monitoring and mass trapping of insect pests. *L. lineolaris* has been shown to use visual and olfactory stimuli in initial orientation to hosts [66,67]. Early studies by Prokopy et al. [67] reported that non-UV reflecting gloss white, yellow, and clear plexiglass rectangles capture more *L. lineolaris* than darker colors. Legrand and Los [68] reported higher trap catch on pink sticky traps than on white traps. Some studies have also investigated the effect of trap design [69,70], height, placement, and time of day [70,71] on the *L. lineolaris* trap capture. Significant differences in trap capture were observed between trapping during late afternoon to dusk hours, using traps placed 20 cm above the ground in cleared areas between two fields compared to other traps placed on the edge or in the center of the field [70]. Fleury et al. [5] reported that weed management practices can affect *L. lineolaris* populations in vineyards, which were captured flying best in traps placed at a height of 40–60 cm. These studies have reported on possible color preferences, trap characteristics, and trap placement, but follow up studies are needed to further optimize these visual cues and develop traps for effective monitoring and management of this cotton pest. Current research studies are focused on optimizing these visual cues and combining them with olfactory and gustatory cues to develop effective monitoring tools for forecasting *Lygus* infestations (personal communication).

### 4.3. Feeding Behavior of Lygus lineolaris

Similar to many other hemipteran phytophagous feeders, *L. lineolaris* also exhibits host selection and feeding site selection behaviors. After landing on the host, the bugs move around, tapping the plant with their antennae and the rostrum [66]. Once a feeding location is selected, stylet probing begins, followed by salivation and re-ingestion of salivated material within various plant tissues [72]. Hagler et al. [73] reported that the average duration of a feeding bout of *L. lineolaris* was over 100 s. Termination of feeding may involve sensory adaptation, central programming, or input from gut stretch receptors [74]. Sevacherian and Stern [75] reported that at 21 °C, 60% RH, and 14:10 photoperiod, *L. hesperus* had intensive locomotor activity at times that corresponds to crepuscular periods under natural conditions. He also found that the *L. hesperus* did not feed during the scotophase, despite extensive probing. Although both *L. hesperus* and *L. lineolaris* primarily rely on herbivory for meeting nutritional needs, they are omnivores and occasionally engage in predation [73,76]. Cannibalism is also common in *Lygus* species, especially in the early instars [77].

Understanding the *Lygus* feeding behavior in meristematic and reproductive tissues of cotton plants is very important as this probing and the associated feeding are the main causes of square abscission, necrosis, and irregular desiccation of pinhead squares. *L. lineolaris* also follows a cell rupture feeding strategy, during which plant cells are lacerated followed by injection of enzymatic saliva and ingestion of liquified plant cell contents through the food canal in the stylets [78]. Using the electrical penetration graph (EPG) recording technique, Cervantes et al. [79] identified and reported the cell rupturing (waveform CR) and ingestion (waveform I) EPG waveforms associated with *L. lineolaris* feeding behavior. This study also reported that the CR phrase triggered the release of tannins, and this phase was positively correlated with the probing activity. Follow up studies have used the EPG technique to examine the effects of Bt expressing cotton (MON 88702) on the feeding behavior of *L. lineolaris* [80]. These studies showed that feeding assays and EPG recordings can be effectively used for screening new Cry proteins against *L. lineolaris*. For further details on the effect of Cry proteins on the *Lygus* feeding behavior, see the host plant resistance strategies section below.

## 5. Insecticide Resistance in *Lygus lineolaris*

Development of pest resistance to conventional synthetic insecticides is a major problem affecting the management of *L. lineolaris* in cotton and other row crops. To maintain pest populations below economic threshold levels, the cotton industry is heavily dependent on seed and foliar insecticidal applications (Figure 3). Current insecticides used for *Lygus* control include contact insecticides (pyrethroids, organophosphates, and carbamates), insect growth regulators such as novaluron, and newer insecticides such as neonicotinoids and sulfoximines that have both contact and feeding activity [81]. Several reviews [82,83,84] and many research articles [42,85,86,87,88,89,90,91,92,93,94] have reported on the chemical control, insecticide susceptibilities, and pesticide resistance of *L. lineolaris,* in laboratory assays and field experiments. Fleming et al. [95] reported the increased use of insecticides in Mississippi Delta at a rate of ~0.2 additional application per year since 1999, reaching an average of five to six applications per year since 2008. Repeated application and prolonged use of insecticides with similar modes of action resulted in the development of resistance by *L. lineolaris*. For example, the organophosphate acephate was commonly used for *Lygus* control in cotton in mid-southern USA, and its prolonged use led to resistant *Lygus* populations. Boll weevil eradication sprays also contributed to organophosphate resistance.

Snodgrass [96] first reported the pyrethroid resistance in *L. lineolaris,* following 14 years of continuous use of pyrethroids for *Lygus* control in Mississippi. Studies by Snodgrass et al. [17,28,40] monitored pyrethroid, carbamate, and organophosphate resistance in *L. lineolaris* populations for over 25 years and provided the rationale for expanded monitoring of pyrethroid resistance given the impact of widespread pyrethroid use on multiple pests. Moreover, they reported that the level of insecticide resistance increases throughout the season, making the *L. lineolaris* more difficult to control in the late season.

Snodgrass and Scott [89] developed a discriminating-dose assay that could detect pyrethroid-resistant *L. lineolaris* populations. Elevated RR_50_ values to pyrethroids (bifenthrin, cypermethrin, fenvalerate, and permethrin) and acephate have been reported using this glass-vial bioassay in the Delta and Hills region of Mississippi [97]. Floral foam feeding bioassays are also used to study the susceptibility of *L. lineolaris* to imidacloprid and thiamethoxam insecticides [86,90,98]. *L. lineolaris* resistance to various classes of insecticides was also studied using atomized sprays to topically treat insects from laboratory and field populations [95,99,100]. Parys et al. [85] used both diet-incorporated assays and glass-vial bioassays to measure the susceptibility of *L. lineolaris* to novaluron. Portilla et al. [81] reported a study that used a combination of different assay methods such as floral-foam bioassays, glass-vial bioassays, terminal bud dip bioassays, and leaf dip bioassays to study the response of *L. lineolaris* to different insecticides such as acephate, imidacloprid, permethrin, sulfoxaflor, and thiamethoxam. Although these different assay methods produced different relative results across the different insecticides, it illustrated the multifaceted activities of these insecticides. Moreover, Portilla et al. [101] developed a solid diet for *Lygus* that has been very useful in screening insecticidal compounds, and this diet has been used for screening orally ingested insecticides [97]. Resistance monitoring assays that are conducted early in the commercial deployment of new insecticides will be very useful in collecting the baseline information on insecticide resistance under field conditions.

Pesticide resistance in *L. lineolaris* to different insecticides is based on a variety of mechanisms [95,102,103,104,105]. Zhu et al. [103] reported that increased resistance to malathion (an organophosphate) was associated with increased carboxylase activity and upregulation of the esterase gene in resistant *L. lineolaris*. Zhu and Snodgrass [104] reported elevated Cytochrome P450 mRNA in pyrethroid-resistant *L. lineolaris* populations. Zhu and Luttrell [105] also reported upregulation of the cytochrome 450 monooxygenase and esterase (EST) genes in imidacloprid-resistant *L. lineolaris* populations. Some field populations of *L. lineolaris* were found to have developed multiple/cross resistance mechanisms to acephate and imidacloprid in some cotton growing areas. Fleming et al. [95], Zhu and Luttrell [102], and Zhu et al. [106] have correlated elevated esterase levels in the Mississippi Delta populations of *L. lineolaris* to reduced susceptibility to acephate. These studies also found variable expression levels of glutathione S-transferase in the Mississippi Delta populations of *L. lineolaris* from 2006 to 2010, and the authors suggested that this change might indicate a potential shift in the genetics of the pest populations. It was also observed that these populations’ susceptibility to organophosphates fluctuated among the months of the growth season, and the level of resistance closely matched the intensity of pesticide use, with the highest resistance levels occurring in October. Laboratory studies by Zhu et al. [104] and Snodgrass [39] reported that esterase and glutathione S-transferase (GST) activities were synchronized with the fluctuation of resistance levels over seasons. Overproduction of esterases causes elevation of metabolic detoxification activity, which leads to the increased organophosphate resistance levels observed in *L. lineolaris*. Recent studies by Dorman et al. [107] reported elevated levels of cytochrome P450 monooxygenase and general esterase (EST) activity in bifenthrin-resistant *L. lineolaris* populations. Jones et al. [93] reported antagonistic effects when bifenthrin and acephate mixtures were used together. Insecticide resistance to commonly used insecticides has been reported in most US cotton growing areas. Novaluron (benzophenyl urea) is an insect growth regulator that acts as a chitin synthesis inhibitor and disrupts molting of immature insects [108]. It is a translaminar insecticide recommended for use against *L. lineolaris* across the mid-southern United States. Even though no control failures have been reported following the use of this product, Parys et al. [85] have reported considerable variability in susceptibility to novaluron within field populations. Efficacy of these insecticides needed to be evaluated early in replicated field experiments with paired testing of the *L. lineolaris* populations using appropriate assay methods to monitor insecticide resistance.

## 6. Integrated Management Strategies

### 6.1. Sampling Methodologies

Sweep nets and drop cloths are commonly used to sample *L. lineolaris* populations. Population estimation methods used for other *Lygus* species are whole plant captures, visual counts, and sampling with suction devices. However, these methods have some, but differing biases and comparisons among them are further confounded by some being relative sampling methods, while others measure absolute densities. Sampling biases associated with sweep net versus drop-cloth methods are important sources of variation to consider when using these tools to estimate *L. lineolaris* populations. Musser et al. [16] found that more *L. lineolaris* adults were collected using sweep nets than by drop cloths, and this greater catch provided a better population estimate of adults. However, drop cloth sampling has been shown to better detect changes in *L. lineolaris* nymphal densities [16,109,110].

Choices of sampling methods and threshold levels vary by time and location in the mid-southern United States. Before peak bloom (squaring period), using a sweep net is the most accurate way to sample for *Lygus* as it helps monitor adult migration into the field [16,111]. After peak bloom, the use of a drop cloth or “beat-sheet” for sampling *Lygus* bugs in cotton is recommended [33] as it provides the ability to monitor nymph densities. During peak bloom, in the last few weeks of effective bloom, *L. lineolaris* are concentrated in the upper portions of the cotton plant or terminal [112]. The terminal contains the remainder of small maturing flowers and squares. Drop cloth counts during this developmental period are likely biased with a lower proportion of the population landing on the cloth further emphasizing the usefulness of plant-based companion measurements. Direct methods for estimating *Lygus* densities include the use of a drop-cloth, sweep net, and from whole plant captures. The beat sheet or “drop cloth” method often employs a white or black drop cloth (91 × 76 cm). The drop cloth is placed in between two adjacent rows and then the canopy above the cloth is vigorously shaken and the numbers of *Lygus* bugs dislodged are counted (sample units are 1.5 row-m per sample). Black drop-cloths are more effective for detecting *L. lineolaris* nymphs, but white shake cloths detect higher numbers of adults [16]. Sweep net samples are generally based on taking 50 sweeps through the top of the canopy with a net 38 cm in diameter. Alternatively, an entire plant can be bagged in a fine mesh fabric or plastic sleeve, clipped, and then taken to the laboratory and frozen before being examined to count *L. lineolaris* adults and nymphs.

Indirect methods of estimating *Lygus* populations are based on measuring damage or density of cotton square. Counts of square abscission or healthy cotton squares are important plant-based methods for estimating the *L. lineolaris* impact. Square retention is measured by inspecting the first position square, which are located on the main stem three nodes below the terminal. The numbers of squares at this location that are present or absent on 50 plants in a plot is the usual measure. Counts may also be made of “dirty squares” out of 25 randomly selected, medium-sized squares per plot [110]. Dirty squares are defined as squares with an external yellow staining caused by the excretions of *Lygus* bugs, which can be recognized under field conditions.

Plant-based sampling methods such as counting of dirty squares and blooms, fruit retention, and the external and internal inspection of young green bolls have been integrated into current IPM strategies for scouting and monitoring them in the field. Sampling for *L. lineolaris* and other plant bugs, however, entails considerable variation across methods and users in addition to being inefficient in terms of resources and time. Gore et al. [32] suggested that these discrepancies may be attributed to variation in the individual sampler or the time lag between *L. lineolaris* feeding and plant response (square abscission). These differences are due not only to the detection method used but are also strongly affected by the personnel taking the samples. Human factors that may explain the discrepancies observed across the plethora of studies and experimental designs include visual acuity, time of day sampling, inconsistent use of sampling technology, and false identification. A correlation exists between sweep net or drop cloth data and the level of square retention, but it entails significant unaccounted sources of variation. Providing proper training to the sampling personnel and using consistent sweeping techniques may help significantly reduce the sampling bias.

Future research towards sampling efforts for *L. lineolaris* may focus on manipulation of plant growth factors such as the removal of reproductive structures at key economic growth stages to experimentally examine how fruit retention and fruit set influence *Lygus* density and distribution. The authors propose that sampling efforts for *L. lineolaris* can be highly accurate, consistent, and efficient under the following conditions: (1) Sampling to be initiated in the mid-morning when crops are dry and free of morning guttation (dew); (2) when ambient and canopy temperatures are optimal for insect activity (i.e., avoiding extreme temperatures); (3) when multiple samplers commence sampling in the same field that users “calibrate” among each other to gauge and standardize the scoring system such that all members are consistently applying the proper methodology.

### 6.2. Sampling Thresholds

*Lygus lineolaris* can damage cotton throughout most of the growing season, but economic damage is most likely to occur during the period from first square through early bloom (pre-bloom), which is the period when feeding on small squares leads to most abscission of cotton squares [32,110,113]. Action thresholds are determined primarily by plant growth stage and the insect density-yield relationship. Thresholds have been defined for *L. lineolaris* in two main physiological cotton growth stages: Pre-bloom and flowering.

Pre-bloom or squaring cotton thresholds are more aggressive and define risk during the more sensitive growth period during the first 2 weeks of square formation. The current sweep net threshold for pre-bloom cotton (first 2 weeks of squaring) is eight plant bugs per 100 sweeps, and higher values trigger control. This level may be lowered if square retention is <85% [16,111]. The current Mississippi drop cloth threshold for *L. lineolaris* during the pre-bloom period of cotton development is 1 *L. lineolaris* per 1.5-row m using a beat sheet [32,111]. As the cotton plant matures, thresholds across all methods generally increase over the remaining 3–7 weeks of bloom as the crop becomes more tolerant to damage.

Flowering (week three of square through the effective end of bloom) thresholds are generally based on drop cloth samples [32,111]. During the flowering stage, *L. lineolaris* thresholds range from eight to 15 bugs per 100 sweeps, or three *L. lineolaris* per 1.5-row m using the beat sheet and 70%–85% square retention [33]. Musser et al. [33] determined that the economic injury level at flowering ranged from 1.6–2.6 bugs per 1.5-row m. Thresholds for *L. lineolaris* during the flowering stages of cotton are based on drop cloth insect counts but may be further refined with the use of plant-based measures such as percent square retention and dirty blooms [16].

Plant-based thresholds have also been established and shown to be a useful supplement to currently accepted thresholds based on insect density for *L. lineolaris* management [32]. Reisig [114] stressed the importance of measuring the weekly square retention rate to determine the extent of *Lygus* bug feeding and impact on abscission. Thresholds for square retention vary slightly among states. Insecticide sprays are recommended when square retention falls below 70%–85% across the mid-southern United States [32,111,115,116]. These various sampling methods may be combined to provide the necessary information to make informed decisions about the need for insecticide applications. The current Mississippi threshold for dirty squares is >10% stained squares, when the sampler is assessing medium-sized squares with exposed buds that have been discolored by plant bug feeding [32,111]. Economic damage from *L. lineolaris* in the mid-south is most likely to occur during the period from first square through peak bloom when plants contain the largest array of susceptible reproductive structures. The combined use of insect-density (i.e., sweeping, beat cloth, visual) and plant-based (i.e., plant mapping, fruit retention, dirty square) sampling methods provide the necessary information to make informed insect and crop management decisions throughout the production season and considered the best management practice for *L. lineolaris* management in the mid-southern United States [16,33,110].

### 6.3. Cultural, Mechanical, and Physical Control

Escalating control costs due to pesticide resistance in *Lygus* populations have created a need for alternative methods of *Lygus* management. Being a highly polyphagous herbivore, *L. lineolaris* populations benefit from diverse landscapes, which allow these plant bugs to increase to high levels in non-cultivated areas on winter and spring annual plant species, from where the bugs later move into fields of cotton, soybean, and corn [7]. Among the many crops that *L. lineolaris* feeds on, it is only considered a pest of cotton in the mid-southern United States.

In a previous review of cultural controls for *Lygus* species, Stewart and Layton [117] discussed the sequence of non-crop host use, and they suggested the possibility of managing *Lygus* bugs on those hosts in order to reduce later infestations in cotton. In a study evaluating the impact of early season applications of the herbicide 2,4-dichlorophenoxy acetic acid (2,4-D) on marginal areas to reduce host plants of *Heliothis* species, the authors also found 65.3% and 72.9% reductions in adult and nymph *L. lineolaris* populations, respectively [118]. More recently, other studies have evaluated the treatment of winter and spring annual host plants used by *L. lineolaris* with selective herbicides in April and March to reduce *Lygus* species reproduction in these habitats [119]. The researchers applied a premix herbicide that included 2,4-D, mecoprop, and dicamba (Strike 3^®^ (Win Field United, Arden Hills, MN, USA), or Trimec ((Gordon’s, Shawnee, KS, USA))) to all non-cultivated areas (roadsides, ditch banks, fallow areas, etc.) within—km^2^ of the study area in the Mississippi Delta. The herbicides applied killed all broadleaf plants but left grasses unharmed. Over the 3 years of the study, the researchers observed a reduction in the number of foliar insecticide sprays needed to control *L. lineolaris* in the herbicide-sprayed area compared to an adjacent untreated control area. Growers in the herbicide-treated area had an average net savings of USD 35,477 per year compared to the growers in the non-treated areas [119]. Research on the use of herbicides to suppress broadleaf plants found that Italian ryegrass, *Lolium multiflorum* (Lamarck), could also serve as an important spring host for *L. lineolaris* in the absence of broadleaf hosts [120]. Italian ryegrass has become an important component of the early to late spring vegetation in the mid-southern United States since it has developed resistance to the herbicide glyphosate in recent years [121]. Glyphosate-resistant Italian ryegrass has also become a serious problem in cultivated fields and field margins. Since it is a grass species and not affected by the herbicides used to suppress broadleaf plants in an areawide approach, the herbicide applications used often released Italian ryegrass from the competition, making it the dominant plant in sprayed non-crop areas in spring. This increase in Italian ryegrass has created a unique challenge for areawide control programs for *Lygus* since it is a reproductive host for *L. lineolaris* [120]. As a result, this management approach for areawide *Lygus* control was not widely adopted by growers. Abel et al. [122] and Cook et al. [123] performed extensive reviews of area-wide suppression of *L. lineolaris* in cotton using herbicides to manage non-cultivated hosts in the spring.

Despite the economic benefits of areawide suppression of non-crop broadleaf plants in spring, the application of foliar insecticides for *L. lineolaris* management has remained an important component of cotton IPM. Nevertheless, various previous studies have demonstrated that factors in the agricultural landscape external to cotton fields can influence *L. lineolaris* populations in cotton. Although *L. lineolaris* is only considered a pest of cotton in most areas of the southern United States, other crops can influence *Lygus* population dynamics. The major field crops in most of the southern United States are cotton, soybeans, and field corn, but rice, peanuts, and grain sorghum can be important in localized regions. Based on previous research, field corn and soybeans appear to be the only crops that significantly affect *L. lineolaris* infestations in cotton. Field corn is generally the first crop planted in the spring followed by soybeans and then cotton. Agricultural consultants often reported greater populations of *L. lineolaris* in cotton adjacent to field corn soon after the field corn has tasseled, and the silks turned brown. Research showed that *L. lineolaris* oviposition and reproduction in field corn is greatest from the tassel stage (VT) to the milk stage (R3) [124] and that field corn is an important driver in the population dynamics of *Lygus* bugs [125]. Soybean production in the southern United States has seen dramatic changes since 2000. Historically, determinate (maturity groups VI and VII) soybean varieties were planted in May and June in the southern United States. More recently, an early soybean production system has been adopted in some regions [126]. This production system utilizes indeterminate soybean varieties (Maturity Groups IV and early V) planted from late-March to early-May. Indeterminate soybean varieties have a much longer flowering period than determinate soybeans, making them attractive to *L. lineolaris* for a longer period. Studies from the midwestern United States [127,128,129] and in the southern United States [130,131,132] have reported *L. lineolaris* in soybean. Only one of those studies [132] reported on the presence of nymphs and evaluated *L. lineolaris* reproduction in soybean. They found that production of new *L. lineolaris* adults on soybean was low, making it a marginal reproductive host [132]. Although reproduction is greater on field corn than soybean, soybean is still an important host in the landscape since the land area planted to soybean is often 2–3× greater than corn in areas where cotton is grown. Proximity of these two crops to cotton can have a significant impact on *L. lineolaris* infestations in cotton. An important consideration for growers when designing crop rotation strategies should be to consider impacts on *L. lineolaris* management in cotton. To minimize *L. lineolaris* migration from other crops, cotton should be planted in large blocks away from corn and soybean rather than as a patchwork where cotton fields are interspersed with other crops. This would minimize the number of edges between cotton fields and other crops and minimize edge effects across an entire farm.

### 6.4. Plant Nutrition and Lygus Infestation

Agronomic crop management practices, apart from pesticide use and variety of choice, can also influence *L. lineolaris* populations. Crop fertility management is an important component of cotton production that must be optimized to maximize yield. In the southern United States, cotton yields are greatest when the rate of nitrogen application is 90 kg N ha^−1^, but growers often exceed this rate in an attempt to increase yields still further [133,134]. Cotton is a perennial shrub with an indeterminate growth habit that is cultivated as an annual crop. Application of too much nitrogen can lead to excessive vegetative growth and plant height, which can delay crop maturity [135]. *L. lineolaris* are highly attracted to lush, vigorous growth on cotton [136], and therefore, high nitrogen application rates can potentially influence *L. lineolaris* movement and management. When nitrogen rates were varied from 45 to 179 kg N ha^−1^, cotton, lint yields were maximized at 90 kg N ha^−1^ [137], in agreement with previous research. In that study, the authors also evaluated the relationship between nitrogen use rates and *L. lineolaris* management needs. They found a positive relationship between nitrogen rate and number of insecticide applications against *Lygus* bug (which were based on current action thresholds) [137]. The average number of sprays was 2.5, 3.0, 3.5, 4.0, and 5.0 for nitrogen rates of 0, 45, 90, 134, and 179 kg N ha^−1^, respectively. Consequently, lint yields and number of insecticide sprays for *L. lineolaris* resulted in profits being maximized at a nitrogen rate of 90 kg N ha^−1^ and economic risk was greater at higher nitrogen rates [137].

A similar trade-off between rates of an agronomic input versus increased management problems with *Lygus* bugs was also observed for the timing of irrigation in cotton [138]. More insecticide applications were needed for *L. lineolaris* control when irrigation was initiated before the first flower (3.6 sprays) compared to when irrigation was initiated at the first flower (1.9 sprays) or peak flower (1.5 sprays). Cotton lint yields were generally similar among irrigation initiation timings except that irrigated cotton produced greater yields than non-irrigated cotton [138]. Cultural practices that promote early crop maturation also shorten the effective flowering period and reduce the number of foliar insecticide applications required for *L. lineolaris* control. Other factors that may affect the timing of cotton maturation, such as thrips injury [139], need to be evaluated to determine their effects on *L. lineolaris* management.

### 6.5. Host Plant Resistance Strategies

Stewart and Layton (2000) reviewed host plant resistance traits among cotton cultivars with a focus on cotton lines that did not produce extrafloral nectaries, also known as nectariless cotton [140,141,142,143,144,145,146]. Nectariless cotton varieties have not been widely adopted in the southern United States. Limited supplies of commercial nectariless varieties have periodically been made available by the Stoneville Pedigreed Seed Co. (Stoneville, MS, USA) and the Phytogen Cotton Seed Co. (Leland, MS, USA). More recently, a public nectariless variety (UA212ne) was released by the University of Arkansas [147].

Other factors associated with cultivar selection for *Lygus* management discussed by Stewart and Layton [117] include trichome density [145,148] and varietal maturation timing [149]. In general, glabrous (few trichomes) cultivars had fewer *L. lineolaris* nymphs than densely pubescent cultivars [143,145]. In a study evaluating the impact of planting date, cultivar maturation timing, and irrigation on the ability of cotton to compensate for early season square loss, an early maturing cultivar that was planted late was less likely to compensate for square loss than a later maturing cultivar planted at any date, or an early maturing cultivar planted early [149]. Irrigation had little impact on the ability of cotton to compensate for manually applied, early season square loss in that study. More recent research has re-evaluated the impact of those factors on *L. lineolaris* management in cotton using natural infestations of *L. lineolaris*. Adams et al. [150] planted two cotton cultivars (early maturing and late maturing) at four planting dates (mid-April, early-May, mid-May, and early-June). Percent yield loss from *L. lineolaris* was greater for the late maturing variety than the early maturing variety, and for the early-June planting date compared to all other planting dates. Both planting date and cultivar affected the number of foliar insecticide sprays needed to maintain *L. lineolaris* densities below the economic threshold [150]. In general, later planting dates required more insecticide applications than earlier planting dates. From a cultivar standpoint, the later maturing cultivar required more insecticide applications than the early maturing cultivar, especially at later planting dates. The authors suggested that the reasons for these results were due to the greater *L. lineolaris* populations later in the season that were more difficult to control [28], and the later maturing cultivar remained at a growth stage susceptible to *L. lineolaris* damage longer than the early maturing cultivar.

In a recent study investigating the impact of leaf trichome density on *L. lineolaris* densities and damage in cotton, no differences were observed in *L. lineolaris* densities among the three cultivars evaluated [151]. The cultivars represented a range of trichome densities including glabrous (56 trichomes per 6.46 cm^2^), hirsute (140 trichomes per 6.46 cm^2^), and very hairy (307 trichomes per 6.46 cm^2^). Although no differences in *L. lineolaris* numbers were observed among cultivars, trichome density did appear to affect the ability of the bugs to feed and damage cotton. During the squaring and early flowering periods, square retention was relatively high for all cultivars. Square retention decreased at a much greater rate on the glabrous cultivar than the hirsute and very hairy cultivars later in the flowering period [151]. As a result, yields were greater for the very hairy variety than the glabrous and hirsute varieties. The authors speculated that small, replicated plots may have affected their results and that research on a larger scale would be needed to determine the value of incorporating higher trichome density varieties into a cotton IPM program. Although densely pubescent cotton cultivars appear to be favorable for *L. lineolaris* management, lepidopteran pests (e.g., *Helicoverpa zea* (Boddie)) prefer to oviposit on leaf substrates with many trichomes [152,153]. Trichome density can also reduce cotton lint quality since more leaf trash adheres to the lint, which then requires additional cleaning. More research is needed to determine the balance between *L. lineolaris* management, lepidopteran management, and lint quality as it relates to trichome densities of cotton cultivars.

Transgenic cotton varieties expressing insecticidal proteins from *Bacillus thuringiensis kurstaki* (Berliner) have been available for commercial production since 1996 [154,155,156]. Since that time, transgenic cottons have been produced that express insecticidal proteins with activity only against lepidopteran pests [157]. Recently, a gene that codes for a novel Bt protein, Cry51Aa2.834_16, has been developed and optimized for control of *Lygus* species [158]. This trait was approved for cultivation in the United States in 2021 [159] under the trade name Thryvon^®^ (Bayer CropScience, St. Louis, MO, USA). Prior to the commercial release, little information was published on this novel Bt trait for *Lygus* management in cotton. Initial studies with transformed plants expressing the TIC807 protein reported 35% to 78% mortality of newly eclosed *L. hesperus* nymphs in the laboratory [160], but no such studies have been published on *L. lineolaris*. Field experiments have shown a reduction in the number of sprays needed for *L. lineolaris* management in Thryvon cotton compared to non-Thryvon cotton [161,162]. Graham and Stewart [161] observed a reduction of 1.25 insecticide sprays for *L. lineolaris* in Thryvon cotton compared to a near isogenic non-Thryvon cotton variety when sprays were made based on the current action threshold. The study found a reduction in total *Lygus* nymphs on Thryvon cotton compared to non-Thryvon cotton, with the primary cause being a reduction in the number of large nymphs. Additionally, yields of Thryvon cotton were greater than non-Thryvon cotton when neither variety was sprayed for *L. lineolaris* throughout the year [161]. A similar study was conducted to compare a 2× threshold to the current threshold and to compare early season benefits to late season benefits [162]. In that study, fewer small (1st–2nd instar) and large (5th instar) nymphs were observed on unsprayed Thryvon cotton than unsprayed non-Thryvon cotton. No differences were observed for medium (3rd instar) nymphs. At the current action threshold (see the Mississippi Insect Control Guide for Agronomic Crops), 1.5 fewer sprays were needed in Thryvon cotton compared to non-Thryvon cotton [162]. This is similar to the 1.25 reduction in the number of applications observed by Graham and Stewart [161], and Thryvon technology is expected to reduce foliar insecticide applications by one to two applications per year. Yields of unsprayed Thryvon cotton were about double that of unsprayed, non-Thryvon cotton. Additionally, yields of Thryvon cotton were greater than non-Thryvon cotton for all spray regime treatments (threshold, 2× threshold, early season only, and late season only), except for the weekly spray treatment [162]. Moreover, yields of Thryvon cotton in the threshold, 2× threshold, and late season only spray regimes were greater than yields in Thryvon cotton that was not sprayed for *L. lineolaris*. These findings suggest that scouting and spraying on current action thresholds in Thryvon cotton will be important to maximize yields and profits for cotton growers. The complete mechanism of action of Thryvon cotton is not completely understood for *L. lineolaris,* but avoidance appears to be an important factor [80,163].

### 6.6. Chemical Control

Chemical control of *L. lineolaris* in cotton was last reviewed in 1999 by Scott and Snodgrass [82]. That review focused primarily on organophosphates, carbamates, pyrethroids, and older classes of insecticides. They also mentioned imidacloprid, the first neonicotinoid labeled for use in the United States. Historically, organophosphates were the most commonly used insecticides for *L. lineolaris* control in southern US cotton [82]. Since this last review, few new insecticides have been labeled in the United States for *L. lineolaris* control in cotton, apart from additional neonicotinoids such as thiamethoxam (Centric, Syngenta Crop Protection, Greensboro, NC, USA). Moreover, since the last review, resistance to neonicotinoids has been reported [86,107]. The benzoylphenylurea insecticide novaluron (Diamond^®^, ADAMA, Raleigh, NC, USA) was registered for commercial use in cotton in 2004 (https://www.farmprogress.com/new-diamond-insecticide-stops-cotton-pests, accessed on 20 June 2021). Novaluron is an insect growth regulator that only affects the immature stages of *L. lineolaris* [164]. However, recent research has shown that novaluron can cause ultrastructural changes in the ovarian tissues of adult *L. lineolaris* that prevent oogenesis and oviposition [165]. Those sublethal effects have also been shown to reduce *L. lineolaris* populations in the field [166]. The registration of novaluron coincided with initial reports of *L. lineolaris* resistance to organophosphates [17]. Novaluron has proven to be a valuable insecticide for *L. lineolaris* management in the mid-southern United States since it provides good control over a longer period than other insecticides. However, considerable variability in the susceptibility of *L. lineolaris* to this product has been measured in bioassays [85], suggesting that it is vulnerable to resistance development.

In 2013, sulfoxaflor (Transform WG^TM^, Corteva Agriscience, Indianapolis, IN, USA) received a federal label from the US Environmental Protection Agency. Sulfoxaflor is a novel insecticide in the sulfoximine class that interacts with nicotinic acetylcholine receptors in susceptible insects [167,168]. Although sulfoxaflor interacts with nicotinic acetylcholine receptors, the sulfoximines were classified as 4C by the Insecticide Resistance Action Committee’s (IRAC) mode of action committee (https://irac-online.org/modes-of-action/, accessed on 6 January 2021). This classification is different in its chemical structure from the neonicotinoids (classified as 4A), and studies evaluating sulfoxaflor against species with known resistance to neonicotinoids suggest that the risk of cross resistance is low [169]. Sulfoxaflor at a rate of at least 50 g ai ha^−1^ provided control of *L. lineolaris* and yield protection similar to acephate, the commercial standard. Efficacy trials in *Arthropod Management Tests* (https://academic.oup.com/amt, search sulfoxaflor and *Lygus*, accessed on 6 January 2021) show similar results. Although several insecticides with different modes of action are currently available for *L. lineolaris* control in cotton, the current state of insecticide resistance development to older classes of insecticides and the potential for resistance to newer classes of insecticides suggests that further development of additional novel insecticides is needed. Insecticides that target unique metabolic processes or physiological systems in insects are needed to add rotational partners to older classes of insecticides that target traditional systems (i.e., nervous system). The Insecticide Resistance Action Committee (IRAC) has classified insecticides into 32 unique modes of action and several additional unclassified modes of action (https://irac-online.org/modes-of-action/, accessed on 9 February 2021). Current insecticides recommended for *L. Lineolaris* control comprise seven of those modes of action. Optimizing other modes of action for control of *Lygus* should be of top priority for the pesticide industry in the future.

### 6.7. Microbial Control

Natural enemies are likely a vital mortality factor of wild *L. lineolaris* populations, but their documented impact can be highly variable. *Lygus* species are attacked by a variety of natural enemies, including predators, parasitoids, and pathogens, though the mirids’ susceptibility varies with life-stage [170,171]. Microbial control has been an important method for managing insect pests, especially using entomopathogenic fungi with contact mode of action [172]. Microbial control of insect pests could diminish the use of chemical pesticides and enhance the environmental safety but currently requires additional research to improve efficacy and economic competitiveness [173]. Since chemical insecticides are not recommended or labeled for mirid or pentatomid insect control on wild host plants, Leland [174] suggested that microbial biopesticides would be particularly appropriate for sucking insect population management. Research on the host range and yearly population build-up of tarnished plant bugs and stink bugs by the USDA-ARS-Southern Insect Management Research Unit (SIMRU) suggests that entomopathogenic fungi are a promising tool for control of hemipteran pests. Species in the genera *Beauveria, Verticillium, Paecilomyces, Metarhizium, Mariannaea, Hirsutella*, *Entomophthora,* and *Isaria* have shown high levels of virulence to *L. lineolaris* under laboratory conditions [175]. Under field conditions, however, just two of these pathogens, *Entomophthora* sp. and *B. bassiana* have been recorded as effective against *L. lineolaris* [176,177]. The most common native pathogen found naturally in North America attacking *Lygus* is *B. bassiana*. Leland and Snodgrass [178] found only 0.3% *B. bassiana* infection in *L. lineolaris* populations from wild host plants in the Mississippi Delta, while McGuire [179] reported a 10% rate of natural infection on *L. hesperus* in California.

The native strain NI8 of *B. bassiana* outperformed any commercially available isolate of the species for control of *L. lineolaris* in terms of insect mortality [174] and suggested the potential for field use [178,180]. The survival of conidia from NI8 in cold storage was higher, and larger production of NI8 made field trials possible. Field trials in cotton of NI8 in combination with novaluron provided control comparable to standard insecticides (acephate, sulfoxaflor) in reducing numbers of *L. lineolaris* adults and nymphs in the cotton [181], but NI8 alone was not tested as a stand-alone treatment. The combined use of mycopesticides and the chemical insecticides is a promising pest control option that needs further attention in management of cotton pests. The synergistic effect of entomopathogenic fungi (EPF) and selective insecticides may increase the efficiency, while minimizing environmental pollution and decreasing the chances of developing resistance to pesticides and EPFs. The compatibility of these EPFs with particular insecticides need to be explored further to develop a suitable IPM and insecticide resistance management (IRM) program for controlling *L. lineolaris* in cotton and other row crops.

### 6.8. Biological Control

Many predators and parasitoids attack *L. lineolaris*. Ruberson and Williams [171] reported 38 natural enemies of *Lygus* bugs in North America, 12 native parasitoids, 5 introduced parasitoids, and 21 native predators. Among these predators, a few species have been studied in terms of their mass rearing by scientists at USDA-SIMRU in Mississippi, namely *Orius insidiosus* (Say) (Hemiptera: Anthocoridae), *Geocoris punctipes* (Say) (Hemiptera: Geocoridae), and *Chrysoperla* sp. (Neuroptera: Chrysopidae) [182]. Field predation rates of these species against *Lygus* bugs have not been measured [170], and in many cases, the insecticides that are toxic to species of *Lygus* are also toxic to their hemipteran predators [170], reducing any impacts of their augmentative release. The efficacy of augmentative biological control of *L. lineolaris* at the field level has not been assessed.

Parasitoids of *L. lineolaris* include the egg parasitoid *Anaphes iole* (Girault) (Hymenoptera: Mymaridae) [171] and the native braconid nymphal parasitoids *Leiophron uniformis* (Gahan) (*Peristenus pallipes* (Curtis), and *P. pseudopallipes* (Loan). In the southwestern United States, populations of *L. lineolaris* can encapsulate *L. uniformis* eggs, with encapsulation up to 70% in the mirid’s second instar [183]. Moreover, *L*. *uniformis* females lay fewer eggs in *L. lineolaris* than any other *Lygus* species. The native parasitoid *A. iole* has a Nearctic distribution, and it is widespread in the United States. However, in the Mississippi Delta, this parasitoid is uncommon in cotton-producing regions [184], but it has been observed on several native host plants. Initial studies on toxicity of field insecticide residues to *A. iole* indicated that it has potential as an inundative biological control agent against *L. lineolaris* in cotton [184]. Although the insecticides tested were highly toxic to *A. iole*, residues of several compounds decayed quickly enough to permit augmentative releases. *Anaphes iole* and the native species of *Peristenus* are the most commonly reared *L. lineolaris* parasitoids. Additional research would be required on effective mass-rearing methods. Cohen [76] developed an artificial diet for *Lygus* spp. that was further improved by Portilla et al. [185], and this diet is effective for mass rearing of *Lygus* species.

Several parasitoids attacking European *Lygus* species have been introduced into the United States, including five braconids: *Leiophron schusteri* (Loan), *P. digoneutis* (Loan)*, P. rubricollis* (Thomson)*, P. nigricarpus* (Szepligeti)*,* and *P. relictus* (Loan) [186]. In the northeastern United States, *P. digoneutis* and *P. relictus* established, but only north of 40° latitude. The USDA introduced *P. digoneutis* in the 1980s for biological control of the *L. lineolaris* in alfalfa [187]. Since its establishment in 1984, it has spread from New Jersey into Pennsylvania, New York, and New England, achieving levels of high levels of parasitism in alfalfa, well above those of the native parasitoids [188,189]. To date, the high impact of *P. digoneutis* on *L. lineolaris* in alfalfa has reduced *Lygus* damage in more sensitive crops such as apples and strawberries [190], and the potential of importing climatically matched *Peristenis* species from Europe for release in more southernly locations in the United States should be investigated further. Tilmon and Hoffmann [191] reported parasitism rates of *L. lineolaris* by *P. digoneutis* and its native congener *P. pallipes* in strawberry fields in New York. They also reported evidence of competition between the two parasitoids, with both showing higher levels of parasitism when the other was not present. Moreover, it was observed that the insecticide usage to control *L. lineolaris* reduced both *P. digoneutis* and *P. pallipes* densities, and the resultant parasitism rates. Another study by Gomez-Dominguez et al. [192] investigated the interactions between the parasitoid *P. relictus* and the generalist *predator Geocoris punctipes* on *L. lineolaris* populations. *G. punctipes* was observed to prey on the majority of immature stages, and preferred 1st and 2nd instar nymphs of *L. lineolaris*, whereas *P. relictus* preferred to parasitize 2nd and 3rd instar nymphs of *L. lineoalris*. Furthermore, *G. punctipes* was observed to prey on parasitized *L. lineolaris* nymphs and the risk of intraguild predation was higher when nymphs contained eggs of *P. relictus*. The simultaneous release of both natural enemies into *L. lineolaris* populations led to increased mortality of 1st, 2nd, and 3rd instar nymphs due to additive effects of predation and parasitism. These results contradict previous studies that reported higher parasitism rates in the absence of a second parasitoid species [191]. These additive effects and intraguild predation factors need to be considered when multiple natural enemies are used for management of pests under field conditions.

### 6.9. Molecular and Genetic Control

To develop molecular tools and genetic strategies for controlling *L. lineolaris*, information is needed on gene expression in the salivary glands and the salivary proteins involved in *Lygus* feeding behavior. *L. lineolaris* engages in “lacerate and flush” feeding behavior with their piercing-sucking mouth parts during which saliva is injected into the plant tissues where it solubilizes cellular and extracellular materials, followed by their ingestion through the stylet. Salivary enzymes play an important metabolic role in food digestion and insect-plant interactions [193]. Pectin degrading enzymes (polygalacturonases, PG) cause plant damage [194] such as square abscission, deformation of squares and bolls, necrosis, reduced vegetative growth, and aborted embryos. Four polygalacturonases (Lhpg1, Lhpg2, Lhpg3, and Lhpg4) are known from salivary gland extract of *L. hesperus* [195,196] and three polygalacturonase encoding genes (Llpg1, Llpg2, and Llpg3) have been reported in *L. lineolaris* [197]. Out of these reported genes, Llpg1 appears to be regulated in response to the current diet [198]. Cooper et al. [199] reported four salivary proteins—laccase, alkyl hydroperoxide reductase-like protein, glucose dehydrogenase, and xanthine dehydrogenase—that may have important roles against plant defense compounds in *L. hesperus*. In a recent study, Showmaker et al. [200] described the salivary transcriptome of *L. lineolaris* and found 28 polygalacturonase proteins, which he grouped into eight PG clades using illumine sequencing technology. Follow up studies by Zhu et al. [201] used cDNA sequencing to identify salivary genes in *L. lineolaris* and found 45 polygalaturonases, two α-amylases, one glucosidase, one glycan enzyme, one aminopeptidase, four lipases, and many serine protease cDNAs. This study also reported 15 serine proteases from the *L. lineolaris* salivary glands that are involved in pre-digestion of plant tissues and inactivation of plant defenses. Previous research has shown that these serine proteases and lipases play important roles in extra-oral digestion, breakdown of plant cell walls, and feeding damage. Improving our understanding of these PG proteins and other salivary gland enzymes can be used for the development of resistant plants through genetic modification and traditional plant breeding methods.

RNAi techniques are a reduced risk approach to the control of pest insects since it can be used to target genes essential for the insect’s growth, behavior, development, or reproduction. Different insect orders respond differently to dsRNA. While coleopterans are highly sensitive to RNAi, hemipterans, dipterans, and lepidopterans exhibit variability in their responses to dsRNA molecules [202]. Walker and Allen [203] described the RNAi-mediated gene knockdown of the *LIIAP* gene (an inhibitor of apoptosis) and its delivery mechanisms in *L. lineolaris.* Follow up studies [203] demonstrated that RNAi-mediated knockdown of IAP correlates to a lethal phenotype in both nymphs and adults when the insects are injected with an IAP effector dsRNA. However, ingestion of this dsRNA did not kill *L. lineolaris* bugs, and experiments showed that *L. lineolaris* saliva can digest this dsRNA and the saliva has a ribonuclease activity that might complicate the use of this RNAi technology for *L. lineolaris* control [204]. Walker and Allen [198] showed that the LlPG1 was the most highly expressed PG gene in *L. lineolaris* feeding on cotton, and this gene could be strongly inhibited (80%) with dsRNA. Targeting this PG gene using the RNAi technique may help reduce damage to cotton plants. Fleming et al. [205] investigated the expression of three PG genes (LlPG1, LlPG2, and LlPG3) in the salivary glands of laboratory and field collected populations of *L. lineolaris*, and they found gene expression differences related to sex, age, region, and the host plant of the *L. lineolaris*. Moreover, it is possible that the selective breeding of host plants with higher polygalacturonase inhibiting proteins (PGIP) might help deter the feeding behavior of *L. lineolaris* and be used as part of a control strategy of *Lygus* management.

Molecular tools are also useful for the study of predator-prey interactions between *Lygus* species and their consumers. Hagler et al. [206] reported a molecular gut analysis technique called universal food immunomarking technique (UFIT) for tagging *L. hesperus* nymphs and adult stages using a protein marker. This UFIT technique combined with enzyme-linked immunosorbent assays (ELISA) identified the different stage-specific predators of *Lygus* species, and this information could contribute to IPM strategies against this pest. Allen et al. [207] reported a new red eye color phenotype of *L. lineolaris* that was caused by a sex-linked mutation, although previous studies reported red eye color in the same species to be due to autosomal mutations. Brent and Hull [208] investigated various eye coloration genes as CRISPR targets and the use of dsRNAs to knock down the eye coloration genes of *L. hesperus*. Genetic studies have also investigated the role of aquaporins, which are membrane channel proteins that facilitate the two-way transfer of water and other solutes across different membranes and play important roles in osmoregulation, excretion, and respiration of insects [209]. Fabrick et al. [210] cloned and characterized five novel aquaporins from the *L. hesperus* species (LhAQP1-5) that play important roles in maintaining water homeostasis. More research is needed to explore the potential to target aquaporins to disrupt osmoregulation and homeostasis in insect pests.

Insect odor binding proteins (OBPs) play an important role in the transfer of odorant molecules and pheromones across the sensillar lymph to the olfactory receptors. Identifying the OBPs that bind with the odorant molecules during this biochemical reaction is an important step in studying the molecular and neural mechanisms involved in insect olfaction. Vogt et al. [211] reported a *Lygus* antennal protein (LAP) with OBP-like properties from the *L. lineolaris.* Hull et al. [212] reported the olfactory co-receptor (*LhOrco*) transcripts in the antennae of adult *L. hesperus* that play an important role in their olfactory perception and odorant discrimination. To further determine the molecular basis of olfaction in *L. lineolaris*, Hull et al. [213,214] identified 33 OBP-like gene transcripts (LylinOBP1-33) using transcriptomic approaches. These OBPs may play a significant role in olfaction as well as gustation as these transcripts were expressed in the antennae and maxillary palps. Hull et al. [215] used homology-based cloning methods and transcriptome data mining to identify guanine-nucleotide binding proteins (G proteins) to elucidate the molecular mechanisms driving signal transduction in *L. hesperus*. Hull et al. [216] recently defined the *Lygus* chemosensory proteins (CSPs) that play roles in chemosensation, development, immunity, and resistance. This study identified 17 CSP-like sequences in *L. lineolaris* and 14 sequences in *L. hesperus*, and these *Lygus* CSPs are orthologues and share sequence identity to previously annotated CSPs from other hemipterans. Further studies may provide more information on the role of these CSPs in the olfaction and chemoreception of *Lygus* pests, which may contribute to development of dsRNA that target olfaction and feeding behaviors.

## 7. Conclusions

Considerable research has been conducted on *L. lineolaris* in the southern US on cotton. The consensus is that *Lygus* infestations and management in cotton is heavily influenced by the surrounding landscape. Wild hosts and alternative cultivated crops, such as corn and soybean, serve as important spring and early summer nurseries where populations build to high densities before moving into cotton. The complexity of *Lygus* management in cotton has been compounded by the development of resistance to multiple classes of insecticides. Since the last review of *Lygus* management in 2000, considerable research has been done to improve and standardize sampling methods and action thresholds across multiple states in the southern US. As a result, the sampling procedures and action thresholds are the same across state lines, especially in states along the Mississippi River. Numerous crop management and IPM strategies have been evaluated for this pest in cotton with varying results. In general, crop production practices that shorten the effective flowering period of cotton can reduce the impact of *Lygus* on yields and reduce the number of insecticide applications required. Some of these practices include, early planting date, early maturing varieties, limiting nitrogen fertilization, and irrigating only when necessary. Additionally, a novel Bt trait that has been developed is expected to significantly improve IPM of *Lygus* in cotton. Despite the IPM approaches currently in use, foliar insecticides remain the most important management strategy in cotton. Novel insecticides with unique modes of action are desperately needed to combat resistance and provide economical control. Given current and projected levels of resistance to pyrethroids, carbamates, and organophosphates in *L. lineolaris*, new environmentally friendly pesticides such as IGRs are needed. The IGRs Novaluron is currently a valuable tool for *L. lineolaris* management in the mid-southern United States since it provides good control over a longer period than other insecticides. However, the restraint use of this insecticide is recommended since there is considerable variability in *L. lineolaris* susceptibility to this compound [85].

The pest status and management of *Lygus* in cotton is directly related to several factors associated with the agricultural landscape. Considerable research has been done to evaluate the ecology of *Lygus* in the southern US, but more research is needed. The lack of an effective pheromone has severely limited the utility of ecological studies evaluating the movement of *Lygus* in the landscape and future research should focus on this aspect of their biology. Transgenic cotton varieties expressing insecticidal proteins have reduced the number of pesticide applications required for control of *L. lineolaris* in Thryvon cotton (Cry51Aa2.834_16) compared to non-Thryvon cotton [161,162]. Bt cotton expressing these Cry proteins need to be tested further to establish their action thresholds for *Lygus* management before releasing them to cotton growers for large scale cultivation. Moreover, recent studies have identified chemosensory proteins (CSPs) and olfactory co-receptors that may provide more information on chemoreception in this phytophagous insect. Identification of new odor binding proteins and salivary transcriptomes show promise of using RNAi mediated gene knockdown technologies to target the olfaction, vison, and feeding behaviors of *L. lineolaris*. More research on biological control agents (parasitoids, predators) and microbials is warranted. Currently, no effective biological control agents suitable for use in the southern United States are available. Research on mass rearing of parasitoids and predators for augmentative biocontrol of *L. lineolaris* is underway. Further efforts on classical biological control, with the goal of finding European species matched to climates in the mid-southern United States is recommended. All these advancements in *Lygus* research in the last two decades has greatly improved our knowledge of the *L. lineolaris* biology, behavioral ecology, population dynamics, host plant resistance, chemical control, and insecticide resistance, and this knowledge could be effectively used for developing behavioral, ecological, cultural, molecular, and genetic tools for use in integrated pest management of *L. lineolaris*.

## Data Availability

Not applicable.
